# The Scottish Diabetic Retinopathy Screening programme

**Published:** 2015

**Authors:** Sonia Zachariah, William Wykes, David Yorston

**Affiliations:** Associate Specialist: Diabetic Retinopathy Screening Service, Glasgow, Scotland.; Consultant Ophthalmologist and Clinical Director: Diabetic Retinopathy Screening Service, Glasgow, Scotland.; Consultant Ophthalmologist: Tennent Institute of Ophthalmology, Gartnavel Hospital, Glasgow, UK. dbyorston@btinternet.com

Diabetic retinopathy (DR) is the leading cause of blindness and visual impairment in the working age population in high-income countries. Since DR is asymptomatic until it reaches an advanced stage, early detection by retinal examination is crucial. This is the goal of a Diabetic Retinopathy Screening (DRS) Programme. In keeping with guidelines for screening programmes, it must adhere to a standardised protocol, have an acceptable standard of sensitivity and specificity, and be amenable to quality assurance at every stage. Simple procedures and easy access for the patient helps to include all those eligible within the population.

The Scottish Diabetic Retinopathy Screening programme was started in 2003. It uses a network of non-mydriatic fundus cameras and specialist software. The software helps with the accurate grading of retinopathy and can generate appointments and referrals to hospital-based eye clinics. It also stores images and patient data.

All eligible patients are invited for photographic screening. First, visual acuity testing is done (using a Snellen chart). Since the fundus cameras are non-mydriatic, dilatation is carried out only if the photographer is unable to obtain an adequate image through an undilated pupil.

The stored images undergo three levels of grading. At Level 1, images with disease are separated from images with no disease. Most Level i grading is done automatically, using a computer programme that detects microaneurysms, but some grading is performed by trainee graders as part of their training.

Level 2 graders are either optometrists or nurse practitioners who have been trained in DR grading. Images with no sight-threatening retinopathy or maculopathy are assigned one of two outcomes:

- return for screening in 6 months
- return for screening in 12 months

Images showing sight-threatening retinopathy or maculopathy which requires referral to hospital eye clinics are sent to Level 3 graders. These are graders are medical retina trained ophthalmologists who are responsible for the fiinal arbitration of referable DR. The Level 3 grader also undertakes internal quality assurance for Level 1 and Level 2 graders, with five hundred random images from each grader included for internal quality assurance each year. All Scottish graders (Level 1 to Level 3) must also participate in external quality assurance once a year, where 100 images are graded over a period of four weeks.

The Scottish Diabetic Retinopathy Screening protocol also includes a list of common non-diabetic pathology, which is detected opportunistically. These are dealt with as per local protocols.

